# MRI-based training model for left atrial appendage closure

**DOI:** 10.1007/s11548-023-02870-w

**Published:** 2023-03-30

**Authors:** Dagmar Bertsche, Mona Pfisterer, Tillman Dahme, Leonhard-Moritz Schneider, Patrick Metze, Ina Vernikouskaya, Volker Rasche

**Affiliations:** Department of Internal Medicine II, Ulm University Medical Center, Ulm, Germany

**Keywords:** LAA closure, Transseptal puncture, Training model

## Abstract

**Purpose:**

Percutaneous closure of the left atrial appendage (LAA) reduces the risk of embolic stroke in patients with atrial fibrillation. Thereby, the optimal transseptal puncture (TSP) site differs due to the highly variable anatomical shape of the LAA, which is rarely considered in existing training models. Based on non-contrast-enhanced magnetic resonance imaging (MRI) volumes, we propose a training model for LAA closure with interchangeable and patient-specific LAA enabling LAA-specific identification of the TSP site best suited.

**Methods:**

Based on patient-specific MRI data, silicone models of the LAAs were produced using a 3D-printed cast model. In addition, an MRI-derived 3D-printed base model was set up, including the right and left atrium with predefined passages in the septum, mimicking multiple TSP sites. The various silicone models and a tube mimicking venous access were connected to the base model. Empirical use of the model allowed the demonstration of its usability.

**Results:**

Patient-specific silicone models of the LAA could be generated from all LAA patient MRI datasets. The influence of various combinations regarding TSP sites and LAA shapes could be demonstrated as well as the technical functionality of the occluder system. Via the attached tube mimicking the venous access, the correct handling of the deployment catheter even in case of not optimal puncture site could be practiced.

**Conclusion:**

The proposed contrast-agent and radiation-free MRI-based training model for percutaneous LAA closure enables the pre-interventional assessment of the influence of the TSP site on the access of patient-specific LAA shapes. A straightforward replication of this work is measured by using clinically available imaging protocols and a widespread 3D printer technique to build the model.

## Introduction

Percutaneous closure of the left atrial appendage (LAA) supports stroke prevention in patients with atrial fibrillation [1–3]. During the procedure, a sheath is advanced through the inguinal vein to the right atrium. Consequently, the atrial septum has to be punctured to implant the occluder in the LAA. Since the anatomical shape of the LAA is highly patient specific, the optimal occluder size [4–6] and the position of the optimal puncture site vary [7, 8]. The huge variability demands patient-specific identification of LAA dimensions and puncture site to avoid repeated device implantation or multiple transeptal punctures (TSP).

For procedure planning, image-based preprocedural assessment of the patient-specific anatomy has gained attention [9–11]. Furthermore, patient-specific 3D printed models have been reported supporting preprocedural planning for device sizing, especially in complex anatomy [12–14]. Thereby, CT-based models were found to be superior to models based on TEE [15], with the disadvantage of increasing the radiation and contrast-agent exposure to the patient. Previous work encouraged using MRI-based 3D printouts in medical applications [16].

In addition to preprocedural assessment, 3D printed models were recommended for the training of interventionalists [17]. However, while the optimal TSP site prediction has already been considered in image-based planning of the LAA closure [18], the consideration of the optimal TSP site in 3D printouts is still missing.

In this work, an MRI-based training model with various predetermined puncture sites and exchangeable LAA was developed to demonstrate the effect of TSP site on the access to the LAA with different shapes and to train how to proceed in the case of a non-optimal puncture site. The models are 3D printed using a widespread 3D printer technique and as such ensures simple replication in different medical facilities. Even though, in its current form patient individualization is restricted to the LAA and a fixed left and right atria model is used, the approach can be utilized as a demonstration tool for patients, medical students and to test innovative closure systems. Further patient individualization is possible by additional 3D printing of the anatomy of interest.

## Methods

The training model includes a single rigid base model comprising the vena cava, right and left atrium including different passages (holes) through the septum, and several interchangeable silicone models of the LAA. The model was derived from 3D MRI patient datasets acquired using a non-contrast-enhanced respiratory navigated 3D isotropic two-point mDixon technique as previously introduced [19]. All data were acquired at a clinical 3T system (Achieva 3.0T, dStream, R5.6, Philips Medical Systems B.V., Best, The Netherlands) with 1.3mm^3^ spatial resolution.

### Base model

**Fig. 1:**
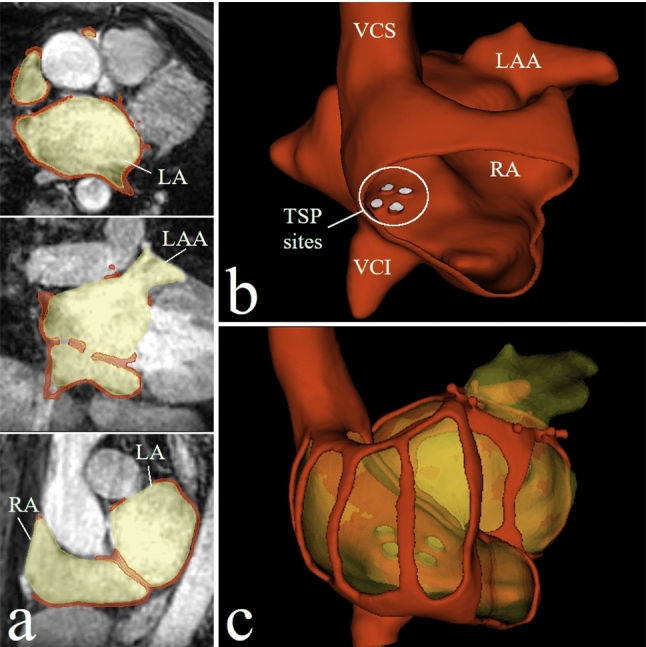
**a** Segmentation (yellow) of the left atrium (LA), left atrial appendage (LAA), and right atrium (RA) based on the underlaying MRI image volume (transversal, coronal, and sagittal plane) with the resulting base model (red). **b** TSP sites in the base model with vena cava superior (VCS), vena cava inferior (VCI), RA, and LAA as landmarks, **c** base model (red) in 3D space with the underlaying segmentation (yellow)

From the MRI volume, the relevant structures were segmented and the respective surfaces were identified using 3DSlicer (www.slicer.org) (Fig. 1a). The relevant structures comprise: left and right atria, LAA, left superior pulmonary vein (LSPV), and vena cava inferior and superior. The LAA was cut out of the resulting surface mesh, and hooks were constructed around the cutout plane to later attach the individual silicone models. At the interface of the vena cava inferior (VCI), a screw connection was inserted. To ensure sight into the inside of the model while maintaining the anatomical shape, additional cutouts were made at the annuli and several other locations of the atria. Based on the MRI data, the area of the fossa ovalis was identified and passages (holes) inserted into the model mimicking different TSP sites using the VCI and aortic root as anatomical landmarks (Fig. 1b). The different TSP sites were placed according to optimal TSP sites for LAA closure as reported in the literature and derived from the retrospective in-house evaluation of 16 cases. TSP sites enabling the delivery of the sheath to the LAA along the axis of the neck of the LAA were considered optimal. Where previously central [20] or inferoposterior [7] TSP sites were recommended, TSP sites located central-posterior (35%), central (24%), inferoposterior (18%), and infero-central (18%) were rated optimal in the in-house evaluation and the four sites were chosen for the base model. The resulting surface mesh of the base model (Fig. 1c) was printed using the widely used fused filament fabrication 3D printing technique with PLA filament (Raise 3D, Irvine, California, USA). Additionally, a tube with a screw connection was printed with 10 mm inner diameter and a length of 350 mm using the same 3D print settings and attached to the approach of the VCI, mimicking the venous access.

### Silicone models of the left atrial appendage

Preprocedural, a 3D MRI dataset was acquired from patients undergoing LAA closure according to the MRI procedure described above. Using 3DSlicer, the LAA and the LSPV were segmented and further processed to generate a cast model with an inner and outer shell. The inner shell corresponded to the initial segmentation. The outer shell had an offset of 2 mm to the inner shell, comparable to the reported thickness of the LAA [21, 22]. The cast model was 3D printed using PLA filament. Subsequently, the printout was filled with silicone (Dragon Skin^®^ 10NV, Smooth on, Macungie, Pennsylvania, USA) with a demolding time of 75 min yielding an elastic modulus of 0.19 N/mm^2^ according to the manufacturer data. The elastic modulus of the silicone is close to the elasticity of heart tissue reported in pigs [23] and as such mimics myocardial tissue properties. After the silicone had dried out, the printed cast model was removed. Finally, small holes were punched out in the silicone model for attachment to the base model with the LSPV using as landmark for the orientation of the LAA.

## Results

The non-contrast-enhanced MRI was sufficiently accurate to enable the segmentation of the relevant structures including the left atrial appendage, left and right atrium, and vena cava. The nominal scan duration for a 3D scan of a patient was 3–4 min with an actual scan duration of ~ 12 min, depending on the heart rate and respiration navigator efficiency.

### Base model

**Fig. 2:**
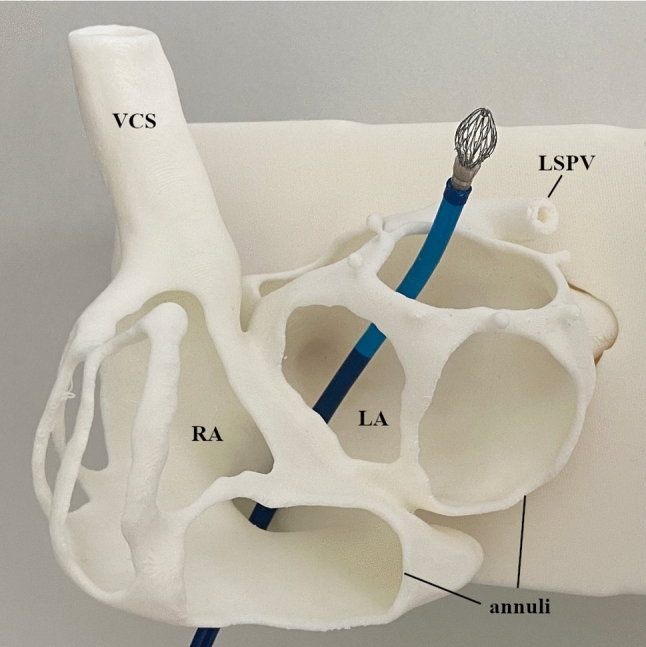
Base model with advanced sheath (Watchman TrueSeal™ double curve, Boston Scientific Corporation, Marlborough, MA, USA) through vena cava inferior, TSP and LAA cutout

Printing of the base model with the anatomical structures, predetermined puncture sites, and attachment hooks for the silicone models was successful within 19 h. The tube mimicking the venous access could successfully be attached to the base model via the screw connection. The sheath could be passed through the cavities of the vena cava, the left superior pulmonary vein, and the puncture sites (Fig. 2). Further, the cutouts allowed a good view into the interior of the model while maintaining stability and the anatomical structure.

### Silicone models

**Fig. 3:**
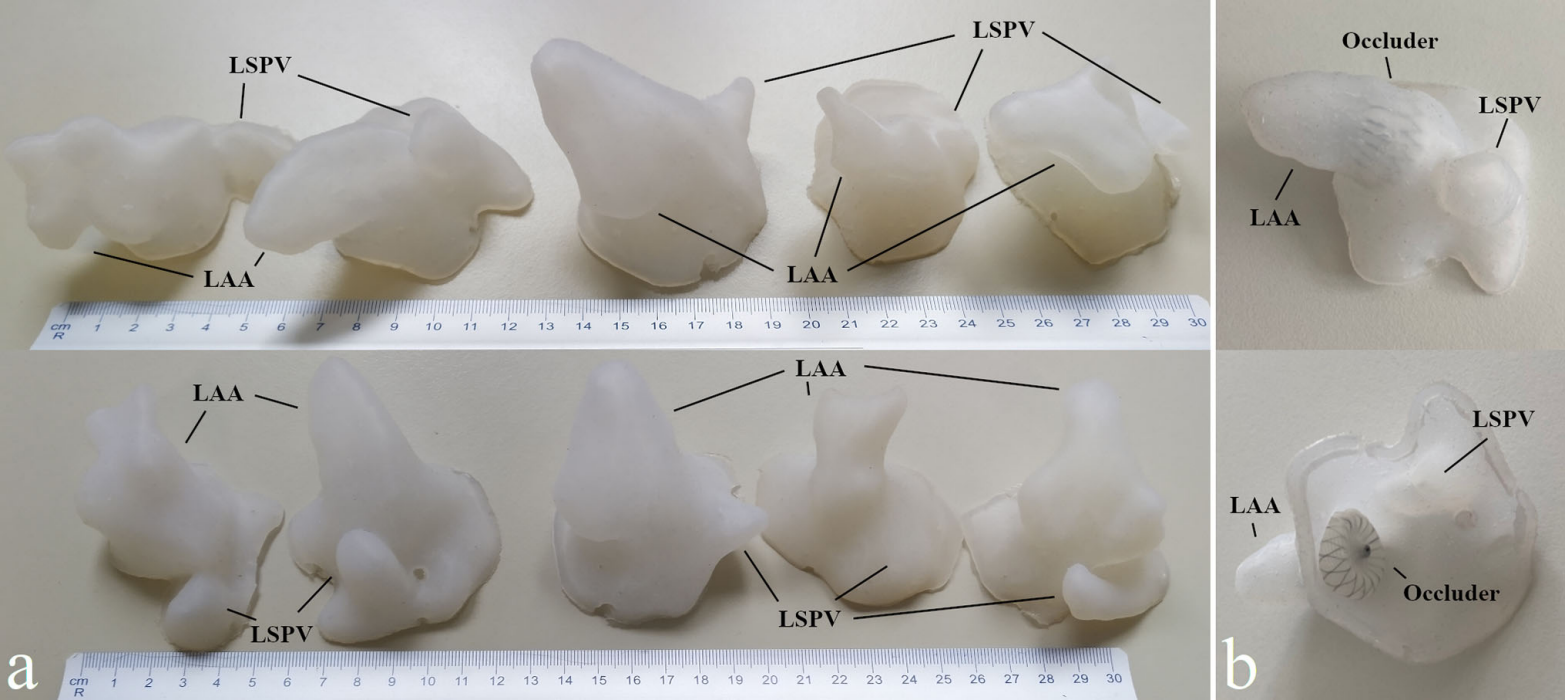
**a** Selection of different silicone models. **b** Clinically implanted occluder (Watchman^FLX^, Boston Scientific, Marlborough, MA, USA) deployed into the respective silicone model

Silicone models of the LAA could be generated from all LAA patient MRI datasets. Figure 3a shows a selection of different silicone models. In each LAA, the respective device as used during the intervention was deployed in the silicone models and visually assessed by the interventional cardiologists. For quantitative validation, the models of two LAA were placed in a water bath after deployment of the occluder and imaged with 3D MRI to derive the respective compression rate of the device in the silicone LAA model. The TEE-derived in vivo compression rate fitted well to the MRI-derived compression rate in the LAA model (mean deviation 2.4% ± 0.9%) and was in good compliance with the clinically recommended range of 10–30%. From the visual inspection (Fig. 3b) and the quantitative analysis, the LAA models appear to match the patient-specific anatomy and the elasticity of heart tissue. The casting models were built using the widely used fused filament fabrication 3D printing technology. On average, the production time of a patient-specific silicone model from the scan to completion took about 6.5 h, with the 3D printing of the cast taking about 4 h.

The impact of repeated device deployment to the silicone model was tested in 2 LAA. The device was deployed 5 times, and after each implantation, an 3D MRI was obtained and the compression rate quantified. Over the repeated deployments, only marginal changes in the compression rate (mean deviation 3.2% ± 2.2%) were observed, showing the stability of the model even during repeated use, e.g., during training.

### Training

**Fig. 4:**
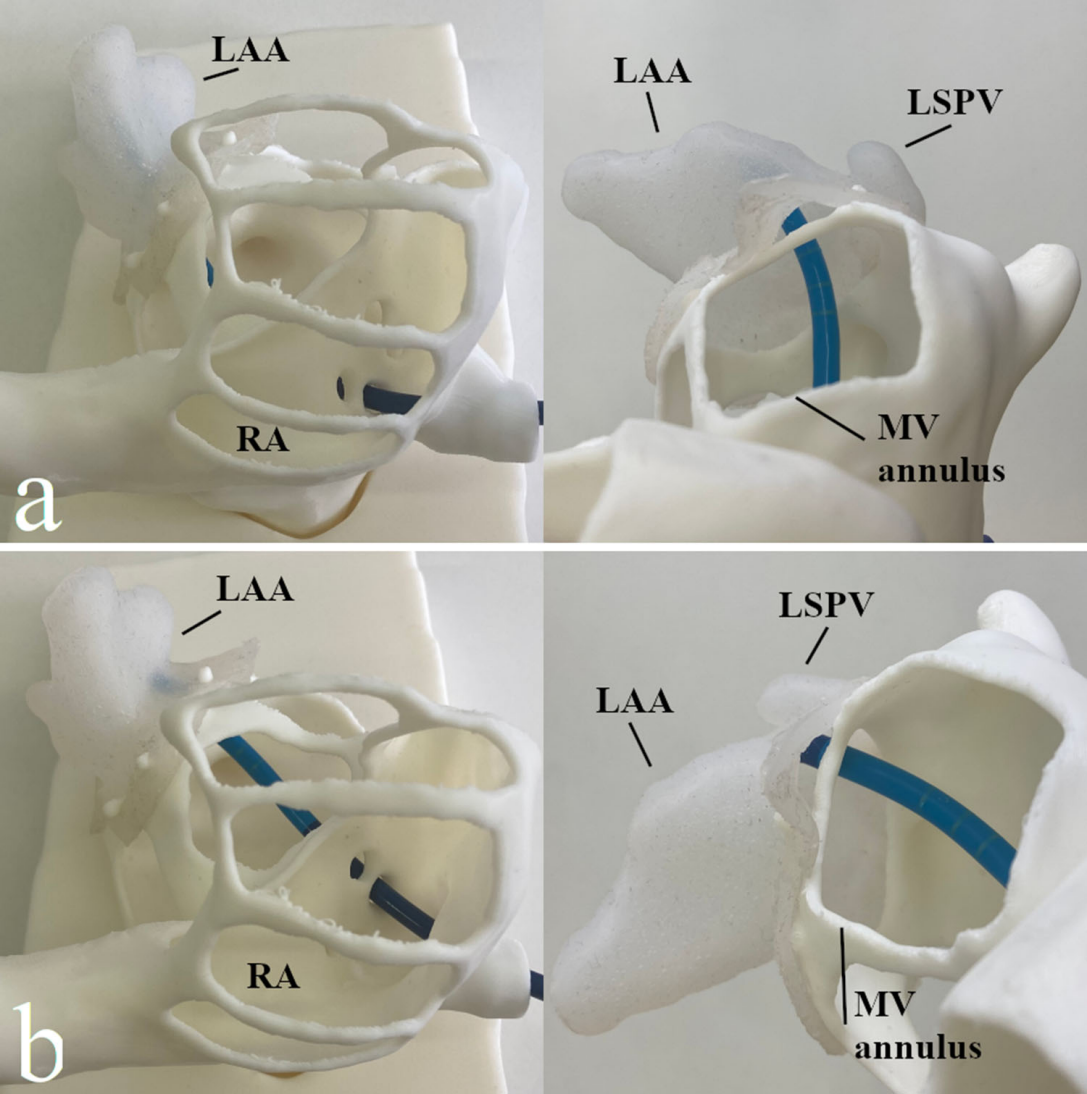
Examples of **a** optimal (sheath delivery to the LAA along the LAA’s neck axis) and **b** non-optimal (sheath delivery not along the LAA’s neck axis) transseptal puncture site in combination with specific LAA shape

All silicone models were successfully attached to the base model. The assessment of the influence of various combinations regarding TSP sites and LAA shapes was possible with a deployment catheter, as exemplified in Fig. 4. Two interventional cardiologists experienced in LAA closure evaluated TSP sites in combination with specific LAA models. For the different LAA, a sufficient central access could be achieved through one of the engrafted transseptal puncture sites. Even using a single base model, variability in the left atrial access route was achieved by the different LAA attachments. Due to the variable transition of the LAA to the atrium and LSPV, the section of the LAA used for LAA model generation was varying, allowing variability in distances and angles between the septum and LAA ostium in addition to shape variability of the LAA. However, if further extended variability or a patient specification is desired, the base model has to be individualized. A complete generation of a patient-specific model without reprinting the detachable tube (MRI scan, base model, and silicone model) would need in total about 26 h, 180 g PLA filament, and 15 g silicone. The tube, mimicking the venous access, enabled the correct transfer of every rotation or movement of the sheath to train the proceeding especially in case of non-optimal TSP. Further, deployment of an occluder could be tested as such demonstrating the applicability of testing occluder systems. Here especially the detachability enabled the accurate assessment the occluder's landing zone.

## Discussion and conclusion

In addition to a sole silicone model of the LAA as commonly used in demonstration/training models, the presented training model offers the possibility to also investigate the influence of the TSP site on the access of patient-specific LAA shapes. The training model clearly shows the impact of the puncture site on the access to the LAA and the associated implantation of the occluder. The added tube mimicking the venous access further enables testing of the correct transfer of the movements to the sheath, providing the possibility of training the proceeding in case of a non-optimal puncture site. In case the model is further individualized and also the currently used base model adapted to the patient-specific situation, the presented approach can be extended to a full preprocedural planning support tool, considering patient-specific atria size, angle of septum wall to LAA ostium, and position of the LSPV. A major limitation here may rise from the expected long 3D printing time resulting in a total model generation time of more than 24 h and the rather high amount of filaments required for printing.

In this work, non-contrast enhanced 3D MRI was used for model generation, providing the required information without any radiation or contrast-agent exposure of the patient. Even though patients were highly arrhythmic, sufficient image quality could be achieved with the imaging protocol and patient-specific models of the LAA could be derived in all cases. Required data for extending the approach to a fully personalized model can be derived from the same dataset. However, a remaining limitation in MRI results from the rather low spatial resolution, which especially in arrhythmic patients may make a detailed analysis of the septum difficult, and using CT instead may be the preferred choice.

The utilization of a widespread 3D printer technique to manufacture both the base and silicone models makes it easy to replicate the construction of patient-specific training models for LAA occlusions enabling a readily accessible demonstration and training tool for medical students, interventionalists, and innovative closure systems. If desired, a more advanced printing technique can also be used to, e.g., include more realistic tissue properties, further reduce 3D printing times of to avoid the need of a cast.

In summary, we have shown that using a single model of the atria in combination with individual silicone LAA models can be used as training models for investigating the impact of different LAA shapes on the TSP. Even though a full planning of the procedure including patient-specific identification of the TSP need further personalization of the base model, already in its current form the silicone model may be applied for devise size selection and at least initial estimates on the optimal TSP. However, the reliability of the patient-specific prediction of optimal TSP site and device size needs to be evaluated in a further study.
